# Pharmacologic treatment of a postpartum ovarian vein thrombosis: Case report

**DOI:** 10.1097/MD.0000000000034711

**Published:** 2023-08-11

**Authors:** Qianqian Gao, Jinqiu Xiong, Hong Jiang

**Affiliations:** a Departments of Obstetrics, Weifang People’s Hospital, First Affiliated Hospital of Weifang Medical College, Weifang, Shandong, China; b Departments of General Surgery, Weifang People’s Hospital, First Affiliated Hospital of Weifang Medical College, Weifang, Shandong, China.

**Keywords:** anticoagulants, case report, pharmacologic treatment, postpartum, venous thrombosis

## Abstract

Postpartum ovarian vein thrombosis (POVT) is a rare disease, and could cause severe complications if ignored. We report a case of POVT, including the patient clinical features, diagnostic methods, treatment, and follow-up results.

**Case presentation:**

A 30-year-old Asian woman was admitted to our hospital because of spontaneous abortion. After the miscarriage, she presented with fever and right lower abdominal pain. A physical examination revealed abdominal tenderness in the right lower quadrant with a palpable mass. Laboratory tests showed leucocytosis and elevated C-reactive protein. Abdominal ultrasound (US) and computed tomography revealed right ovarian vein thrombosis (OVT). The patient was treated with systemic anticoagulation and antibiotics and was discharged 22 days later on a regimen of an oral anticoagulant. 1.5 months after discharge, an US with a color Doppler examination showed no OVT.

**Conclusion:**

A high index of suspicion is required in cases of abdominal pain and fever after delivery, especially if unresponsive to antibiotics. It should be differentiated from acute appendicitis, accessory abscess, endometritis, ovarian torsion, and other acute abdominal diseases. For a POVT case with a definite diagnosis, drug treatment may be effective enough.

## 1. Introduction

Postpartum ovarian vein thrombosis (POVT) is a rare venous thromboembolic disease with a prevalence of 0.05% to 0.18%.^[1]^ The first case of POVT was reported at the beginning of the 20th century. Women with this condition often present with nonspecific symptoms, such as an unresponsive fever, pelvic pain, or abdominal pain. The diagnosis is frequently made after a woman fever is nonresponsive to antibiotics after 48 hours. However, the diagnosis is often complicated by the other nonspecific signs and symptoms reported by patients: back pain, nausea, vomiting, tachypnea, tachycardia, hypotension, ileus, and sepsis. The differential diagnosis includes endometritis, appendicitis, adnexal torsion, broad ligament hematoma, adnexal abscess, sepsis, pyelonephritis, and septic pelvic thrombophlebitis. The thrombus may extend to the inferior vena cava (IVC) or renal vein, even causing pulmonary embolism. If not treated in time, fatal complications may occur.^[2]^ This case is a presentation of ovarian vein thrombosis and illustrates why this diagnosis should be considered in women presenting postpartum with fever and abdominal pain.

## 2. Case presentation

A 30-year-old woman (gravida 2 para 1) was admitted to our emergency department with fever and regular uterine contractions at 23-week gestation. Initial physical examination showed mild fever (37.4°C), obesity (body mass index, 29.6 kg/m^2^),^[3]^ abdominal tenderness, and negative rebound tenderness. Her pulse was 102/min. Her first pregnancy concluded a live birth. She had no significant medical history, denied smoking, and denied a family history of venous thromboembolism (VTE). According to the Caprini risk assessment tool,^[3]^ her total VTE risk score was 2 points (pregnant and obesity), which is indicative of low risk. Laboratory examination showed the following results: leukocytosis (17.08*10^9^/L) with neutrophilia (15.15*10^9^/L) and elevated C-reactive protein level (111.7 mg/L).

**Figure 1.**
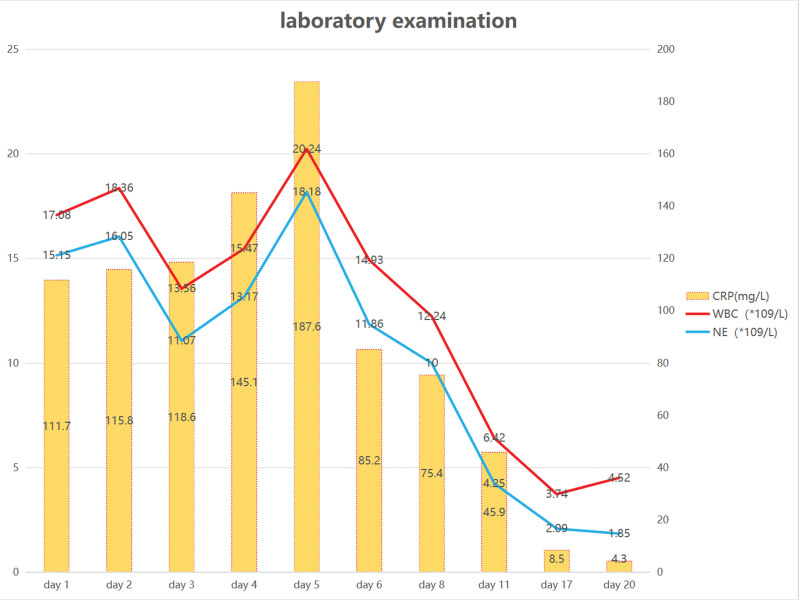
Laboratory examination.

**Figure 2.**
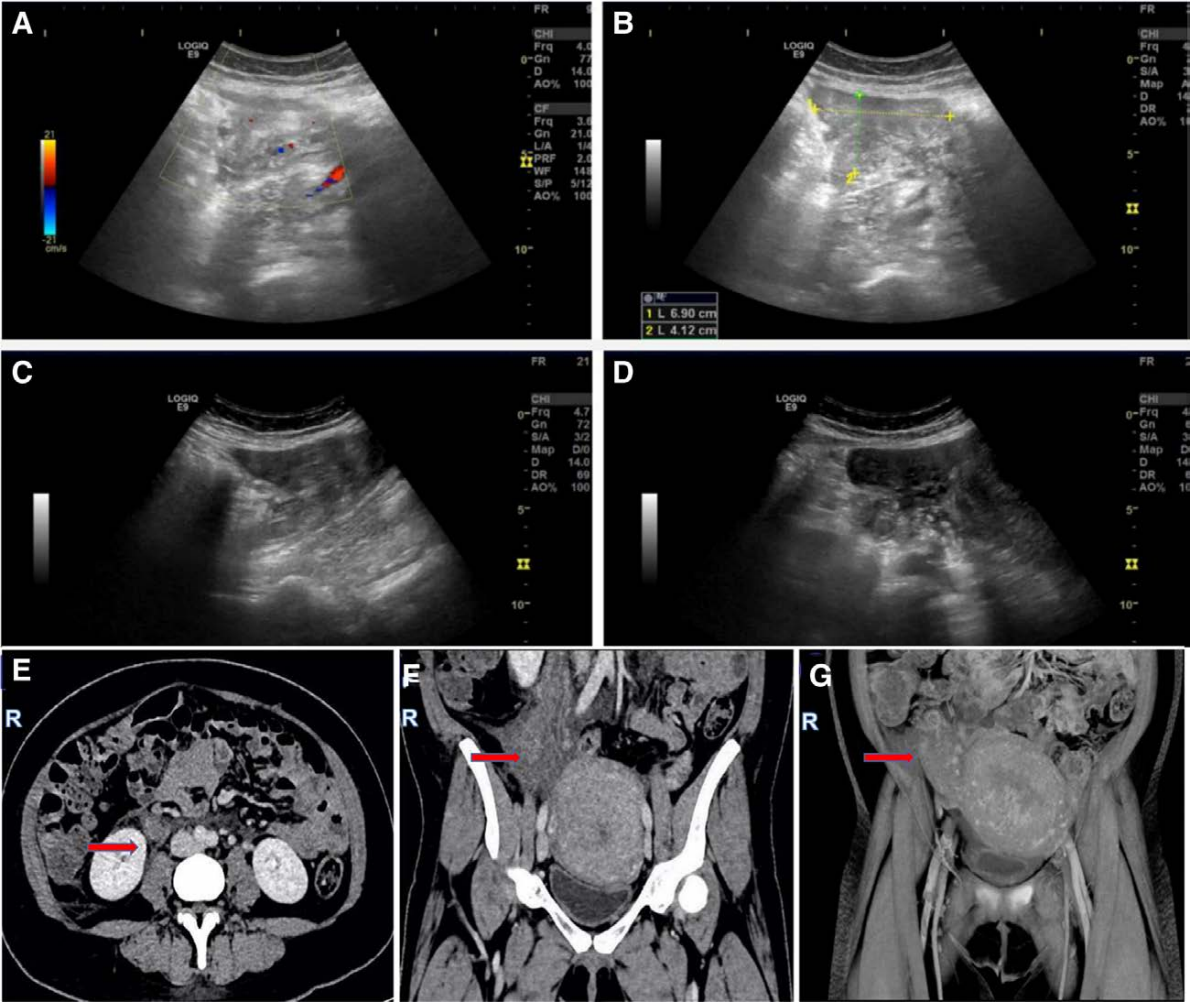
Imaging examination. Day 3 morning US (A and B) 6.9 * 4.1 cm hypoechoic area was detected above the right ovary, with irregular shape, unclear boundary, and a little blood flow signal. Day 3 afternoon US (C and D) 7.2 * 5.3 cm hypoechoic area was detected in the right accessory area, with boundary was unclear and rich blood flow signal, surrounding ovarian. Tubular anechoic structure with thick wall and poor internal sound transmission was detected on the outside. Day 3 CT (E–G) Right ovarian vein thrombosis (The arrow indicates the lesion). US = ultrasound.

**Figure 3.**
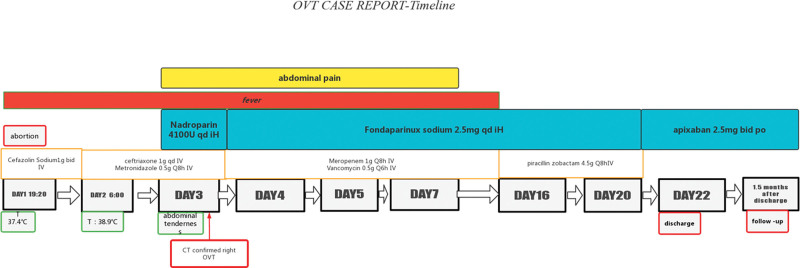
Timeline of postpartum ovarian vein thrombosis (POVT).

Spontaneous abortion happened on the night of admission. Dilatation and curettage procedure was performed because of retained placenta, followed by antibiotics therapy (cefazolin sodium,1 g bid iv), because of the cause of abortion does not exclude intrauterine infection. After the miscarriage, the patient reported severe fever (temperature 39.5°C) and chill. Laboratory tests revealed leukocytosis (18.36*10^9^/L) with elevated neutrophilia (16.05*10^9^/L) and C-reactive protein level (115.8 mg/L) (Fig. 1). Based on laboratory results, the patient was diagnosed with a bacterial infection. Since infection increases the risk of VTE (postpartum = 1, miscarriage = 1 and obesity = 1), the patient was placed on low molecular weight heparin (LMWH) (Nadroparin,4100AXaIU Qd iH) for anticoagulation therapy as prophylactic dose 2 days after abortion. The patient complained of abdominal pain after 3 days of persistent fever; a physical examination indicated right lower quadrant abdominal tenderness with positive rebound and guarding. A mass of 8 cm in diameter could be palpable in right lower quadrant abdomen. Pelvic ultrasound (US) revealed an inflammatory mass on the right side. (Fig. 2). The initial diagnosis includes appendicitis, adnexal abscess, and septic pelvic thrombophlebitis. Abdominal CT revealed right OVT (Fig. 2). Due to refractory fever, the patient was initiated on antibiotic treatment with meropenem (1 g Q8h) and vancomycin (0.5 g Q6h). As CT confirmed the existence of right ovarian vein thrombosis, the dose of anticoagulant was changed from prophylactic dose (Nadroparin, 4100AXaIU Qd iH) to therapeutic dose (Fondaparinux sodium, 2.5 mg Qd iH). Anticoagulation therapy with LMWH (Fondaparinux sodium, 2.5 mg qd) continued until discharged. The patient abdominal pain symptoms lasted for 4 days, while the fever symptoms disappeared after 15 days, with normal laboratory test results. And the patient was discharged on postpartum day 22. LMWH was switched to oral anticoagulation (apixaban 2.5 mg bid). 1.5 months after discharge, no abnormality was found in pelvic ultrasound. The patient refused to continue anticoagulation therapy. To date (1 year after her delivery), no adverse event has been reported during follow-up. No readmission took place (see the timeline for more details) (Fig. 3). Pathology of abortion tissues revealed chorioamnionitis. Doppler US excluded deep vein thrombosis of the lower limbs. All the interventions were tolerated well. No heparin-induced thrombocytopenia and abnormal hemorrhage occurred.

## 3. Discussion

POVT is a rare but serious thrombotic disease, and the misdiagnosis and underdiagnosis was a common occurrence because of its nonspecific clinical features.

### 3.1. Ovarian vein anatomy and pathophysiology

The ovarian vein plexus is connected with the uterine vein plexus, making pelvic infections easy to spread. The right ovarian vein flows into the IVC at an acute angle, resulting in invulnerability to vascular pressure fluctuations; the left ovarian vein flows into the left renal vein at right angles. Ovarian vein diameter increases in pregnancy; a thrombus is formed in the valve area when the flow of blood changes. POVT could be explained by Virchow triad. The dextrorotation of the gravid uterus compresses the IVC, increasing the venous pressure significantly and leading to pelvic vein stasis. Increased circulation of clotting factors and soaring estrogen levels during pregnancy result in a hypercoagulable state in the postpartum period. Production of bacteria and proinflammatory cytokines affects the coagulation system by inducing tissue factors if there is local inflammation invasion, and coagulation activation is accompanied by functional impairment of major anticoagulant mechanisms.^[4]^ Most POVT cases involved the right ovarian vein, according to previous reports.^[2]^ It occurred less frequently on the left or bilaterally. POVT occurred more commonly after cesarean section, according to previous literature.^[2]^

### 3.2. Risk factors

Risk factors included puerperal surgery or procedure, twins pregnancy, postpartum hemorrhage, premature labor,^[5]^ infection,^[2]^ premature rupture of membranes,^[5]^ diabetes^[6]^ and blood transfusion, and hypertension,^[5]^ were also mentioned. In our reported case, multiple risk factors existed.

### 3.3. Symptoms and physical examination

The typical presentation of POVT was acute pelvic and abdominal pain or fever, most frequently occurring within 10 days after delivery, and the pelvic and abdominal pain typically localizes to the side of the affected vein.^[5,6]^ Other clinical manifestations could include nausea, vomiting, chill, waist pain, and so on. Abdominal pain can radiate to the groin, iliac fossa, flank, or lateral abdomen. Antibiotic treatment is usually ineffective. Physical examination presented peritoneal signs, such as abdominal-pelvic tenderness, positive rebound tenderness, and guarding.^[6]^ Sometimes palpable abdominal mass could be found.^[5]^ Our patient initially complained of fever and abdominal pain with abdominal tenderness, positive rebound tenderness, and guarding, which were typical characteristics.

### 3.4. Diagnosis and differential diagnosis

Differential diagnosis is crucial, including endometritis, acute appendicitis, acute pyelonephritis, urinary obstruction, accessory torsion, accessory abscess, hematoma of the broad ligament of the uterus, and other acute abdominal diseases. Patients with POVT may undergo laparotomy due to acute abdominal manifestation (suspected of accessory torsion or appendicitis).

At present, imaging examination is the mainstay diagnostic method. CT has high sensitivity and specificity. The typical imaging finding is the presence of the retroperitoneal tubular structure anterior to the psoas muscle with a filling defect, which is a central round low-attenuation center and peripheral higher attenuation rim.^[1]^ Secondary signs were inhomogeneously enhancing parauterine mass, besides an enlarged uterus. Inexperienced radiologists may ignore the above imaging findings. The limitation of CT examination is that it exposes the patient to ionizing radiation, and if there are contraindications, such as renal insufficiency and contrast agent allergy, a CT examination cannot be performed. US combined with Doppler examination has advantages such as portable examination instruments (bedside examination is feasible), lower cost, contrast agent not required, no radiation exposure, and easily carried out in a grass-root medical unit. The formation of other pelvic vein thromboses can be checked. The sensitivity and specificity of US examinations are lower than those of CT and magnetic resonance imaging (MRI).^[7]^ On the US scan, OVT appeared as a tubular structure with thickened veins, central filling defect, no Doppler blood flow signal, or a mass with no echo or low echo between the accessory area and the IVC, and no blood flow signal.^[6]^ However, the US efficiency may be hampered by the operator experience and methods, obesity, or gaseous bowel distention. MRI angiography shows low signal intensity (acute) or high signal intensity (subacute) on T1-weighted imaging, while an intermediate-high signal intensity at the center, surrounded by peripheral low signal intensity edges on T2-weighted imaging.^[8]^ Because of the examination is expensive and difficult to obtain in some medical institutions, we recommend CT as first line and MR as alternative. MRA applies to cases where other examination methods cannot diagnose POVT.

None of the POVT-related laboratory tests demonstrated high sensitivity and specificity. It is remarkable that elevated infection-related indicators, such as leukocytosis, elevated CRP, elevated D-dimer level, and anemia, were observed, according to previous literature. Our patient blood culture was negative, but Gram-negative bacteria were cultured from the vaginal smear. Thrombophilia workup may be positive,^[2]^ but the positive rate also was low. Our test results were all negative, including antithrombin III lupus anticoagulant, antinuclear antibodies, anticardiolipin antibodies, anti-double-stranded DNA antibodies, serum immunoglobulin G production, beta-2 glycoprotein antibodies, lupus anticoagulant, and serum SSB/SSA antibody profile; however, plasma protein C activity decreased (76%, evaluation criteria 82%) while protein S was normal. Remarkably, POVT occurred in our patient, even though her VTE assessment indicated low risk, and she received anticoagulation therapy after delivery. D-dimer levels increases in pregnancy with a hypercoagulable state, leading to lower specificity for POVT diagnosis in the postpartum period.^[9]^

### 3.5. Treatment

It remains unclear if treatment with antibiotics and anticoagulation leads to resolution of the clinical findings (pain, fever) or merely allows time for the patient to improve on their own. Some patients received broad-spectrum antibiotics combined with anticoagulation or thrombolytic therapy^[5,6]^; some patients recovered on anticoagulation therapy alone or accepted surgery, such as the placement of an IVC filter.^[6]^ Anticoagulant therapy included LMWH and oral anticoagulant drugs. The duration of treatment was often 3 months, but supporting statistical analysis is unavailable due to incomplete data. During the follow-up period, the thrombus dissolved or alleviated in above patients, the clinical symptoms were alleviated, and the prognosis was benign. All patients had no thrombotic recurrence, bleeding, or readmission during the follow-up period.

Prior studies failed to reach a consensus regarding the best treatment of POVT, except for 2 recommendations that POVT should be treated with conventional anticoagulation for 3 to 6 months (1C)^[10]^ or 1 to 3 months (III-C).^[11]^ Some research showed no statistically significant correlation between treatment and no treatment with anticoagulation in terms of overall outcomes unless there was pelvic sepsis.^[12]^ The incidentally detected role of anticoagulation treatment for POVT is controversial.^[12]^ Our patient underwent antibiotics and anticoagulation treatment with rapid improvement and had no recurred VTE or bleeding. It is safer for lactating women to use LMWH because oral anticoagulants may be excreted in breast milk. Patients on heparin/heparinoid products should be counseled on heparin-induced thrombocytopenia. Oral anticoagulants are classified into vitamin K antagonists and non-vitamin K antagonist oral anticoagulants. A retrievable suprarenal IVC filter is a viable treatment option for patients who fail to respond to anticoagulation or in whom anticoagulation is contraindicated. Surgery (such as pelvic vein ligation and surgical thrombectomy) is applicable to patients with recurrent pulmonary embolism, drifting thrombosis, and systemic anticoagulation treatment failure.^[13]^

Broad-spectrum antibiotics are recommended for patients diagnosed with OVT with suspected infection or those with fever and should be continued 48 hours after the fever and clinical symptoms improve (II-2A); if sepsis or complex infection is combined, a longer antibiotic therapy course is necessary (III-C).^[11]^

Complications included an extension of the thrombus into the vena cava and/or renal veins, according to previous studies.^[2]^ POVT could induce urinary obstruction, causing hydronephrosis and kidney failure.^[13]^ Fatal complications included pulmonary embolism, dyspnea, loss of consciousness, shock, and cardiac arrest.^[14]^ Pelvic congestion syndrome and recurrent deep vein thrombosis were noted in POVT.^[15]^

## 4. Conclusion

OVT remains and enigmatic entity, often a diagnosis of exclusion with empiric therapy. POVT should be highly suspected when a patient has abdominal pain or fever within 10 days after delivery, especially when there is a history of perinatal surgery or uterine cavity operation, twin pregnancy, postpartum hemorrhage, premature delivery, premature rupture of membranes, etc. Prompt and early diagnosis is critical. It is recommended to conduct a CT examination in time. Pressure US combined with Doppler examination can be used for screening and dynamic monitoring. MRI is an alternative. Laboratory tests can assist in the differential diagnosis. Once POVT is diagnosed and the risk of hemorrhage is ruled out, anticoagulation therapy with drugs is feasible. The program should be individualized, and the treatment duration should be 3 months after delivery. Patients with definite infections should be placed on broad-spectrum antibiotic treatment promptly.

The limitation of this article is that our case reports were retrospective, making it hard to establish causality. The superiority of this case report lies in the detailed and complete record of the patient course of the disease, examination, and therapeutic schedule, while this approach will not replace an RCT, it might provide clues about what avenues of further study might be most productive, providing the basis for subsequent clinical research. Based on the rarity of POVT, most of the available documents were case reports without randomized controlled studies; there were only 1 multicenter retrospective study and a prospective study at a single medical center, with limited numbers of cases. The treatment guidelines for OVT are all from the diagnosis and treatment guidelines for VTE. No high-quality POVT treatment guideline can be adopted. Identification and treatment in the early phase can reduce the prevalence of complications and mortality. Considering that the diagnosis of POVT needs to be combined with clinical manifestations, imaging, laboratory tests, and treatment, including drug and surgical interventions, it requires multidisciplinary cooperation among obstetrics, imaging, laboratory, and general surgery departments in a medical center. In the future, well-designed randomized controlled trials need to be set up to establish the diagnosis and treatment guidelines for POVT.

## Acknowledgments

We are grateful to Xuwen Yang and Zhengyu He for supporting for the authors work.

## Author contributions

**Resources:** Hong Jiang.

**Writing – original draft:** Qianqian Gao.

**Writing – review & editing:** Jinqiu Xiong.
